# Delivery of luminescent particles to plants for information encoding and storage

**DOI:** 10.1038/s41377-024-01518-x

**Published:** 2024-08-28

**Authors:** Wei Li, Junjie Lin, Wanyi Huang, Qingrou Wang, Haoran Zhang, Xuejie Zhang, Jianle Zhuang, Yingliang Liu, Songnan Qu, Bingfu Lei

**Affiliations:** 1 Key Laboratory for Biobased Materials and Energy of Ministry of Education, College of Materials and Energy, South China Agricultural University, 510642 Guangzhou, China; 2 Joint Key Laboratory of the Ministry of Education, Institute of Applied Physics and Materials Engineering, University of Macau, 999078 Macau, China; 3 Maoming Branch, Guangdong Laboratory for Lingnan Modern Agriculture, 525100 Guangdong Maoming, China

**Keywords:** Imaging and sensing, Nanoparticles

## Abstract

In the era of smart agriculture, the precise labeling and recording of growth information in plants pose challenges for modern agricultural production. This study introduces strontium aluminate particles coated with H_3_PO_4_ as luminescent labels capable of spatial embedding within plants for information encoding and storage during growth. The encapsulation with H_3_PO_4_ imparts stability and enhanced luminescence to SrAl_2_O_4_:Eu^2+^,Dy^3+^ (SAO). Using SAO@H_3_PO_4_ as a low-damage luminescent label, we implement its delivery into plants through microneedles (MNs) patches. The embedded SAO@H_3_PO_4_ within plants exhibits sustained and unaltered high signal-to-noise afterglow emission, with luminous intensity remaining at approximately 78% of the original for 27 days. To cater to diverse information recording needs, MNs of various geometric shapes are designed for loading SAO@H_3_PO_4_, and the luminescent signals in different shapes can be accurately identified through a designed program, the corresponding information can be conveniently viewed on a computer. Additionally, inspired by binary information concepts, MNs patches with specific arrangements of luminescent and non-luminescent points are created, resulting in varied luminescent MNs arrays on leaves. An advanced camera system with a tailored program accurately identifies and maps the labels to the corresponding recorded information. These findings showcase the potential of low-damage luminescent labels within plants, paving the way for convenient and widespread storage of plant growth information.

## Introduction

The evolution of traditional agriculture into precision agriculture, driven by the advent of information technology and the Agricultural Internet of Things (IoT), marks a significant shift in modern farming practices^1,2^. Precision agriculture harnesses real-time monitoring, data collection, and intelligent decision support to enhance agricultural efficiency and environmental conditions^2^. In the pursuit of building a smart farm, it is necessary to label and record various life indicators of plants for comprehensive monitoring^3,4^. Conventional plant labeling methods involve hanging PVC waterproof tags on plants, but these are susceptible to damage, disorder, and loss. Therefore, the development of an intelligent plant labeling system that ensures reliability, simplicity in recording, reading, and updating, and seamless integration with the IoT network platform are imperative.

Long afterglow materials exhibit prolonged luminescence and a high signal-to-noise ratio property^5^, which make them promising candidates for innovative luminescent plant labels with new functional attributes. Among numerous luminescent materials, SrAl_2_O_4_:Eu^2+^,Dy^3+^ (SAO) stands out as a leading long-afterglow material, known for its strong and long-lifetime afterglow luminescence along with excellent ultraviolet resistance^6^. Despite significant research focusing on rare earth ion-doped strontium aluminate materials and performance enhancement, the poor water resistance of SAO remains a critical challenge limiting its practical application^7^. The strong polarity of water causes SAO to be vulnerable to hydrolysis, thus requiring effective encapsulation methods to preserve its phase structure^8^. While various encapsulation measures, such as SiO_2_^9^, TiO_2_^10^, MgF_2_^11^, and polymeric capsulation layer^9^ have been explored, many sacrifice luminescent performance, hindering the material’s application potential. Therefore, the exploration of encapsulation techniques that enhance both water resistance and luminescent performance is crucial for establishing SAO as an excellent luminescent material for constructing plant labels.

**Fig. 1:**
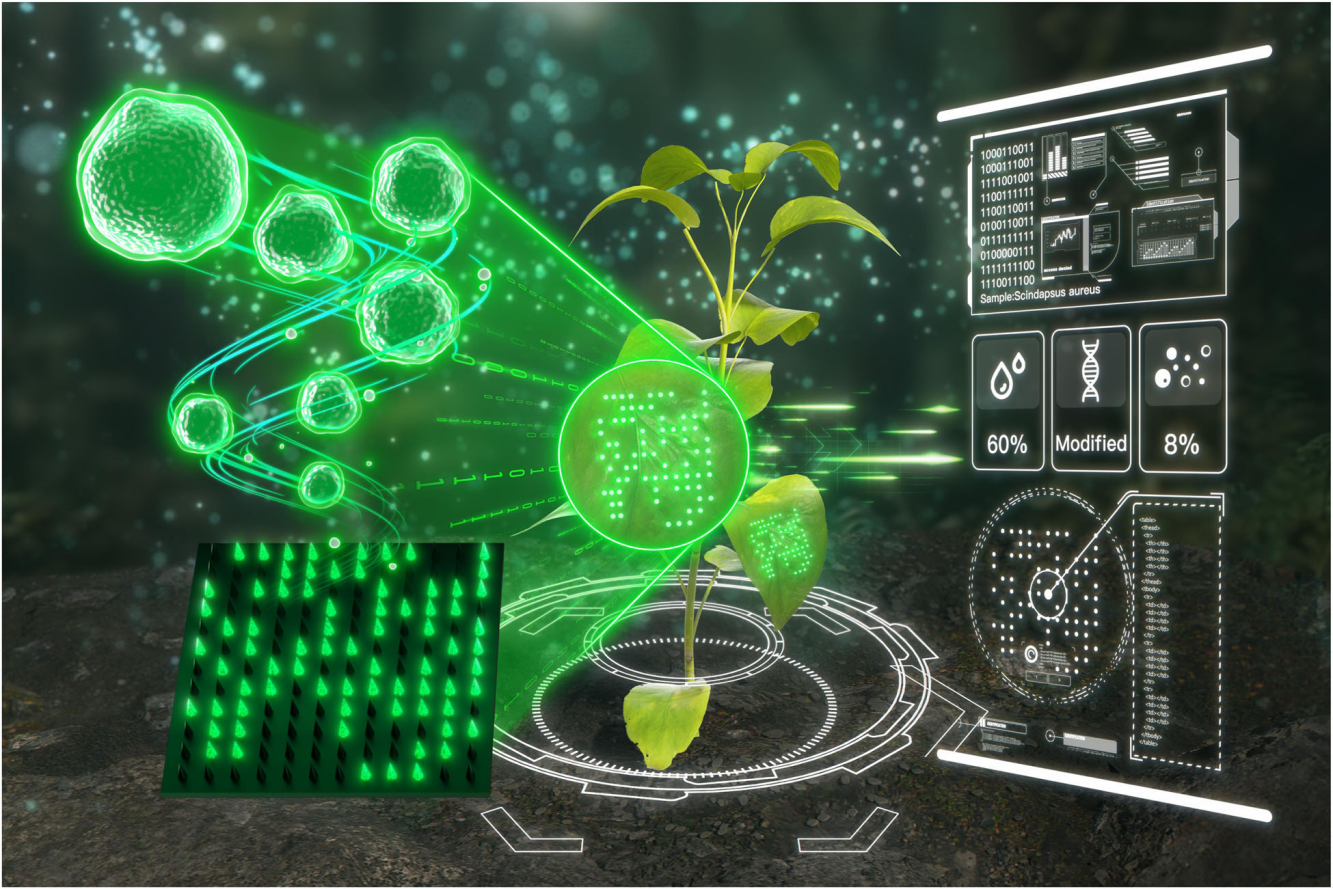
A schematic diagram illustrating the construction of a plant information cloud platform using luminescent plant labels

In the previously reported work, commonly employed methods for delivering materials into plants include foliar spray^12,13^, root absorption^14^ and trunk/petiole injection^15,16^. However, these methods have limitations in delivering micron-sized particles into plants. While the trunk/petiole injection method can directly enter the vascular system through mechanical damage to the cuticle and epidermis and other barriers, its invasiveness makes it only suitable for some large woody plants. In recent years, MNs patches, used in drug delivery applications for their minimal invasiveness, safety, and efficiency, present a promising alternative^12^. In a previous report, near-infrared luminescent particles were injected into the skin using microneedle patches to record information on vaccine administration over a long period^17^. Drawing inspiration from MNs patches in the medical field, we employ them for delivering long-afterglow materials into plant leaves to achieve information recording and encoding. For the construction of an information recording platform in plants (Fig. 1), SAO is first encapsulated with H_3_PO_4_ to enhance water resistance and maintain stable luminescence within the complex internal environment of plants. The tips of MNs patches are then loaded with SAO@H_3_PO_4_ to create well-arranged luminescent arrays, providing plants with specific encoded information. The compilation of this information aims to establish an intelligent agriculture platform wherein plant luminescence labels serve as gateways to cloud platforms for storing various physiological information and realizing a precision agriculture system based on IoT.

## Results

**Fig. 2:**
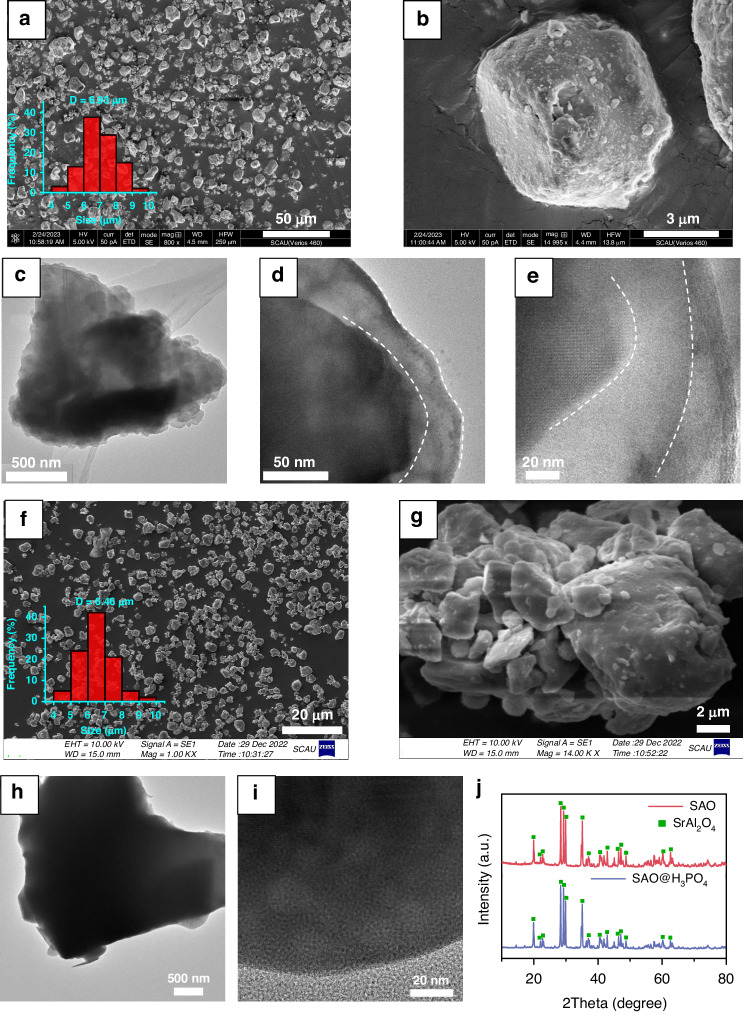
The morphological characterizations of SAO and SAO@H_3_PO_4_. SEM (**a**, **b**), TEM (**c**, **d**), and HRTEM images (**e**) of SAO@H_3_PO_4_; SEM (**f** and **g**), TEM (**h**), and HRTEM images (**i**) of SAO; (**j**) XRD patterns of SAO and SAO@H_3_PO_4_.The insets in (**a**) and (**f**) show the size distribution of particles

The morphology of SAO was characterized by transmission electron microscopy (TEM) and field emission scanning electron microscopy (SEM). As shown in Fig. 2, the average particle size of SAO@H_3_PO_4_ is 6.93 μm which is irregularly shaped with rough surfaces (Figs. 2a, b, and S1), while the average particle size of SAO is 6.46 μm and its surface present a relatively smooth state (Fig. 2f, g). The above results indicate that the encapsulation of H_3_PO_4_ has slightly increased the particle size. Furthermore, from the TEM images in Fig. 2c, d, it can be observed that the SAO@H_3_PO_4_ has a translucent encapsulating layer at the boundary, with an average thickness of ~30 nm. However, a clear crystal lattice stripe is observed at the boundary of pristine SAO (Fig. 2h, i), indicating that H_3_PO_4_ encapsulating forms a translucent protective layer on the surface of SAO. From the HRTEM images, it is noted that after H_3_PO_4_ encapsulating, there is no crystalline lattice in the encapsulating layer, indicating that the layer is amorphous (Fig. 2e). In contrast, the edge of SAO is well crystallized with clear crystalline lattice (Fig. S2). Moreover, a similar crystalline lattice is observed in the phosphors inside the encapsulating layer (Fig. 2e), indicating the successful encapsulating of H_3_PO_4_^18^. Subsequently, the phase of the samples was analyzed with an X-ray diffractometer and the XRD patterns of SAO@H_3_PO_4_ and SAO are shown in Fig. 2j. Characteristic peaks corresponding to spinel strontium aluminate are seen in the XRD patterns of both samples, notably at 2*θ* values of 28.58°, 29.38°, and 29.98°^19,20^. In common, SAO tends to hydrolyze and deteriorate upon contact with water. As shown in Fig. S3, the pH value of SAO suspension in water reaches 11.8 in 1 day and 12.5 in 7 days, while for SAO@H_3_PO_4_, the pH value is 6.0 in the first day and keeps stable around 6.4 in 7 days, benefiting from the encapsulation of H_3_PO_4_ for the phosphors. These results show that H_3_PO_4_ treatment could form a protective layer of SAO and could not change the phase of phosphors.

**Fig. 3:**
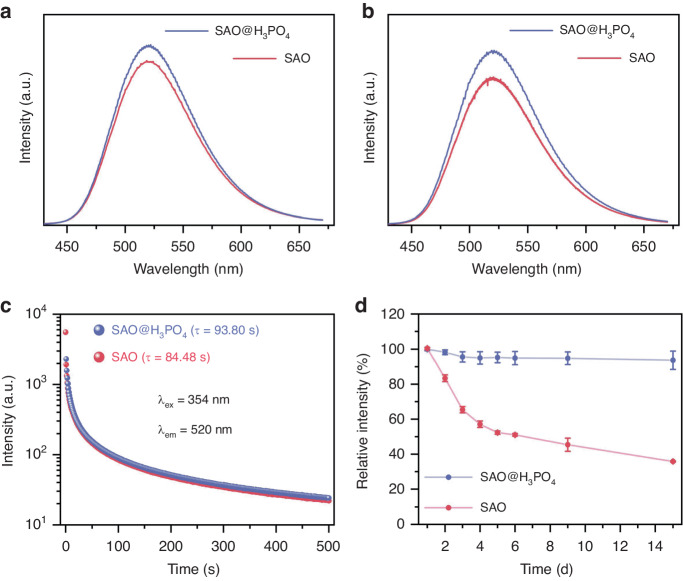
The optical properties of SAO and SAO@H_3_PO_4_. Fluorescence emission spectra (**a**), phosphorescence emission spectra (**b**), and phosphorescence decay curves (**c**) of SAO and SAO@H_3_PO_4_. **d** Fluorescence emission intensity changes of SAO and SAO@H_3_PO_4_ immersed in juice extracted from the plant

In order to explore the optical properties of SAO@H_3_PO_4_, the fluorescence spectra, phosphorescence spectra, and phosphorescence decay curves were recorded. From the fluorescence spectra of SAO and SAO@H_3_PO_4_ (Fig. 3a, b), we found that the strongest emission of both peaked at 520 nm, which is derived from the 4*f* → 5*d* transition^21^. Compared with SAO, the emission peak of SAO@H_3_PO_4_ does not show an obvious shift. It is noteworthy that SAO@H_3_PO_4_ shows enhanced optical properties in terms of luminescence intensities and phosphorescence lifetime (Fig. 3a, c). According to previous work, the enhanced luminescence performance is due to the existence of amorphous encapsulating layers. When photons are irradiated on SAO@H_3_PO_4_, the amorphous encapsulating layers have a low refractive index^22^, which reduces the number of refracted photons and increases the number of absorbed photons, thus leading to enhanced luminescence^23^. Then, in order to explore the stability of SAO@H_3_PO_4_ in plants for further applications, we immersed SAO and SAO@H_3_PO_4_ in the juice extracted from the plant and measured their luminescence spectra, respectively. Luminous changes of SAO and SAO@H_3_PO_4_ in plant juice at room temperature are plotted in Fig. 3d. During the 2-week observation period, the fluorescence intensity of SAO shows a decrease of about 60%, while the SAO@H_3_PO_4_ maintains a high fluorescence intensity (>95%). In general, H_3_PO_4_ treatment not only improves the luminous properties of SAO but also improves its luminous stability in plants. H_3_PO_4_ treatment gives SAO the potential as long-afterglow materials for luminescent labeling in plants.

**Fig. 4:**
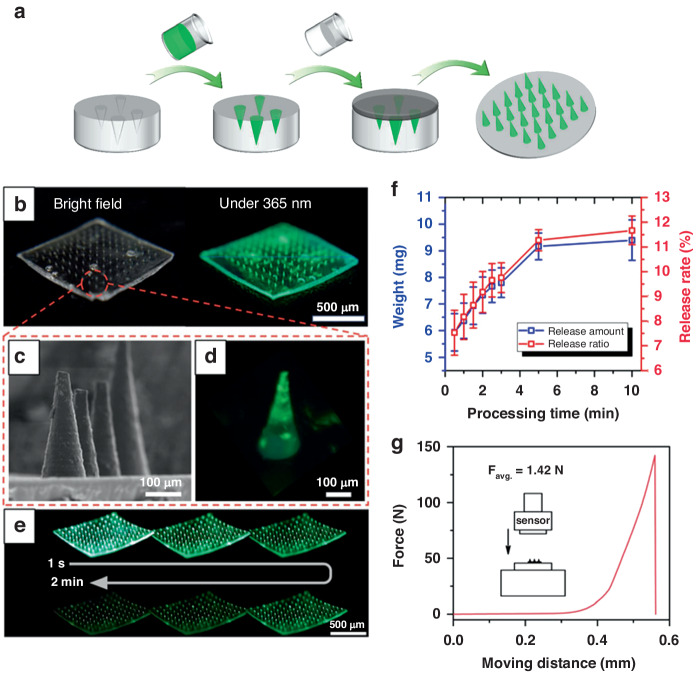
The characterizations of SAO@H_3_PO_4_-loaded MNs patch. **a** Diagram of preparation process of SAO@H_3_PO_4_-loaded MNs patch. **b** Electronic images of SAO@H_3_PO_4_-loaded MNs patch at ×20 magnification under white light and a 365 nm lamp, respectively, **c** SEM image of SAO@H_3_PO_4_-loaded MNs patch. **d** Electronic image of a single tip of SAO@H_3_PO_4_-loaded MNs patch at ×60 magnification under a 365 nm lamp. **e** Phosphorescent images of SAO@H_3_PO_4_-loaded MNs patch are recorded every 24 s after removing the 365 nm excitation. **f** Release curve of sodium hyaluronate matrix loaded with SAO@H_3_PO_4_. **g** Compression curve of the SAO@H_3_PO_4_-loaded MNs patch

In order to transport SAO@H_3_PO_4_ into plants, a new injection method using MNs as carriers was explored. The SAO@H_3_PO_4_-loaded MNs patch can be readily constructed by a micro-molding method, as schematically illustrated in Fig. 4a. In this method, the MNs patch is made of sodium hyaluronate, which has strong mechanical strength after drying. Meanwhile, it has high water solubility, allowing it to rapidly dissolve and quickly release the loaded SAO@H_3_PO_4_ upon contact with water^24^. As shown in Fig. 4b, the MNs patch shows a 10 × 10 MNs array with a base diameter ≈ 180 μm, height ≈ 350 μm, and center-to-center space ≈ 1000 μm. The SEM image of a single tip of the SAO@H_3_PO_4_-loaded MNs patch shows a sharp conical tip (Fig. 4c). The fluorescence images show that the green fluorescence signal mainly comes from the tip of the MNs patch, indicating that SAO@H_3_PO_4_ is mainly loaded at the tip (Fig. 4d), which shows bright and visible phosphorescence by naked eyes after the removal of excitation source (Fig. 4e). When MNs patch contacts with water, the loaded SAO@H_3_PO_4_ can be rapidly dissolved and delivered into plant tissues. The relationship between the release amount and dissolution time of loaded SAO@H_3_PO_4_ was studied (Fig. 4f). Within 0–5 min, the dissolution rate is fast, and the release amount increases rapidly. Within 5–10 min, the dissolution gradually tends to balance, and the release rate slows down. The loading amount of SAO@H_3_PO_4_ was evaluated by dissolving the MNs in the water, and the result reveals that the loading capacity is 45.57 ± 5.69 μg per MNs patch. The mechanical strength of the SAO@H_3_PO_4_-loaded MNs patch was measured under compression (Fig. 4g), and the results show that the breaking point of each needle is 1.42 N, which is higher than the reported minimum effective force required to pierce the plant tissue (>1 N per needle)^25^, thus confirming its potential ability to pierce the plant tissue and its scalability for in vivo applications.

**Fig. 5:**
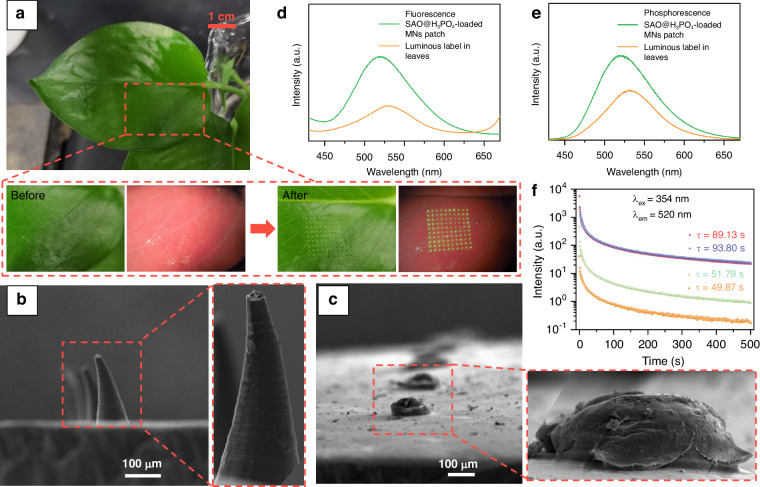
The characterizations of SAO@H_3_PO_4_-loaded MNs patch before and after injection. **a** Digital photograph of leaf before and after the injection of SAO@H_3_PO_4_-loaded MNs patch under white light and 365 nm UV lamp, respectively; SEM images of SAO@H_3_PO_4_-loaded MNs patch before (**b**) and after (**c**) injection; Fluorescence (**d**) and phosphorescence (**e**) spectra of SAO@H_3_PO_4_-loaded MNs patch and leaf with SAO@H_3_PO_4_ label. **f** Phosphorescent decay curves of SAO (red line), SAO@H_3_PO_4_ (blue line), SAO@H_3_PO_4_-loaded MNs patch (green line) and leaf with SAO@H_3_PO_4_ label (orange line)

We then tested the ability of the MNs patch to deliver SAO@H_3_PO_4_ into plant samples. Because the plant leaves are thin and soft, the MNs patch can easily pierce the leaves under a small pressure. After maintaining the needle tip in the leaves for 1 min (Fig. 5a), the apparent green fluorescence of the SAO@H_3_PO_4_ array can be observed on the leaf surface, indicating that the transfer of loaded SAO@H_3_PO_4_ from the needle tip to the plant leaf surface occurs. The SEM images of the MNs patch show that the MNs present a sharp conical shape before injection (Fig. 5b), and only a blunt MNs base remains after injection (Fig. 5c). The results show that when MNs were injected into the plant leaf, the tips of the MNs were largely dissolved by the plant juice to realize the transportation of SAO@H_3_PO_4_ from the MNs patch to the plant leaf^26^. The injected leaf was characterized by SEM, and as displayed in Fig. S4, the particle size of SAO@H_3_PO_4_ is nearly unchanged within the leaf. To investigate the transport behavior of SAO@H_3_PO_4_ delivered to the plant, the Sr element content at different locations of plants after injection for 1, 14, and 27 days was tested using inductively coupled plasma (ICP) spectroscopy. From the results in Fig. S5, it is evident that the Sr element contents are notably elevated at the injection sites. In contrast, the Sr element contents in the leaf tip and stem areas remain largely consistent with that of the blank group, indicating that the SAO@H_3_PO_4_ delivered to the plant does not undergo further transportation within the plant once it reaches its initial destination. This also ensures that the constructed label can maintain its morphology for a long time. The optical performance is a fundamental property of luminescent labels, so the luminescence spectra of SAO@H_3_PO_4_-loaded MNs patches and the realized SAO@H_3_PO_4_ within the leaf surface were compared. As shown in Fig. 5d–f, there is no obvious shift in the luminous peak position, indicating that the detected green optical signal comes from SAO@H_3_PO_4_, which was delivered to plant leaves. However, the luminous intensities and lifetime detected on the leaf surface of the plant have significantly decreased, this is because only SAO@H_3_PO_4_ particles loaded in the tip part of the MNs enter the plant during the delivery process, the remaining loads at the bottom of the MNs resulting in the reduction of the detected luminous intensities and phosphorescent lifetime in leaves. On the other hand, after SAO@H_3_PO_4_ particles are delivered into the leaves, the photons emitted by SAO@H_3_PO_4_ need to penetrate the epidermal cells of the leaves to be detected/observed, which is also a reason for the decrease in luminous intensities. Nevertheless, the fluorescence and afterglow performance of SAO@H_3_PO_4_ can still provide strong enough optical signals to be collected by high-definition cameras as luminescent labels embedded in plants.

**Fig. 6:**
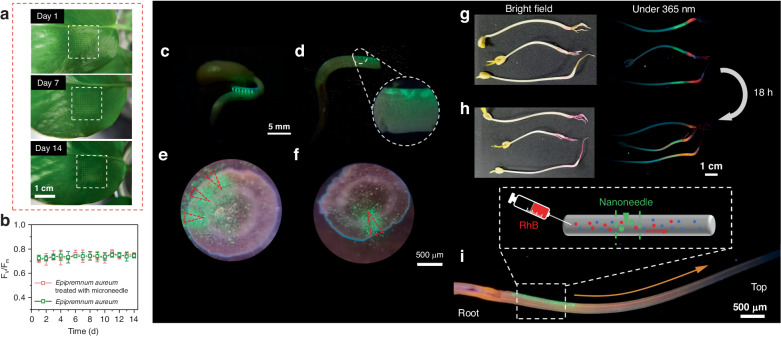
The physiological characterizations of the plants after injection. **a** Digital photograph of the leaf after injection within 14 days. **b** Photosynthetic efficiencies of SAO@H_3_PO_4_ injected and untreated leaves within 14 days; Longitudinal (**c** and **d**) and cross-section (**e** and **f**) images of injected bean sprouts under 365 nm UV light; Images of plants after delivery of SAO@H_3_PO_4_ and RhB (**g**) and continued cultivation for 18 h (**h**) under white light and 365 nm light. **i** Longitudinal image of bean sprouts co-cultured with SAO@H_3_PO_4_ and RhB for 18 h. The inset is a schematic diagram of the experiment

The injection behavior and the impact of SAO@H_3_PO_4_ on plant health were investigated based on the study conducted by Cao et al. ^27,28^ From the aspect of phenotype, in Fig. 6a, there is no obvious scar tissue around the injection site after 14 days. In addition, the photosynthetic efficiency of leaves with and without SAO@H_3_PO_4_ injection was compared in 14 days, the test was only conducted on the injected parts of the leaves, which shows negligible difference compared to the control group (Fig. 6b). Furthermore, we also tested other physiological indicators of SAO@H_3_PO_4_ injected leaves within 7 days, such as actual photosynthetic efficiency, electron transfer rate, and non-photochemical quenching (Fig. S6). During the testing period, no significant changes are observed in the physiological indicators of the plants, and the differences between the experimental and control groups are not significant. However, a slight increase is observed in the non-photochemical quenching of the experimental group, which can be attributed to the interference of the fluorescence emitted by mechanical damage caused by injection behavior^29^. These results indicate that the injected SAO@H_3_PO_4_ particles have no obvious adverse effect on plant health. On the other hand, during the growth process, plants will continuously absorb water from the environment to meet the needs of normal life activities. So, the sap flow of plants is a vital behavior in life activities. However, the presence of foreign particles may cause the interruption of plant sap flow, so Rhodamine B (RhB) was selected to mark water molecules to test the sap flow in the plant after injection. Bean sprout was chosen as the experimental model because the transparency of their stems facilitates observation, and their autofluorescence is relatively weak. In Fig. S7, SAO@H_3_PO_4_ was first delivered to the stem of the plant with MNs patch, and the clear injection site and fluorescence signal were observed under a 365 nm UV lamp. The longitudinal section images (Fig. 6c, d) and the transverse section images (Fig. 6e, f) show that the MNs patch successfully delivered SAO@H_3_PO_4_ to the vascular system of the plant. Then, inject the RhB solution below the SAO@H_3_PO_4_ injection position, which is closer to the root. After 18 h, the orange-red fluorescence signal of RhB was observed to fill the stem of bean sprouts, and a clear sap flow path was observed in the longitudinal section of the stem (Fig. 6g–i). The flow of RhB along the stem can be inferred that SAO@H_3_PO_4_ injection will not affect the sap flow in the plant. In conclusion, the MNs patch-based injection strategy for SAO@H_3_PO_4_ delivery does not show adverse effects on plant growth or in vivo substance transport. Furthermore, we conducted an investigation into the potential movement of embedded SAO@H_3_PO_4_ particles within plants toward their surrounding environment. Following a 7-day observation period, fluorescence spectra of water samples and soil environments in which the plant grew were recorded (Fig. S8). The spectra show no presence of green fluorescence, indicating the absence of SAO@H_3_PO_4_ particles in the surrounding environment. These results imply that once SAO@H_3_PO_4_ particles are embedded within plants, they stay localized at the injection site and do not undergo transport with the plant fluids into the surrounding environment. To further test the biocompatibility of SAO@H_3_PO_4_, an MTT assay on the HeLa cell line was performed (Fig. S9), suggesting the good cell viability of SAO@H_3_PO_4_ with up to 1000 mg/L, and the dosage of SAO@H_3_PO_4_ injected into plants is far less than 1000 mg/L. These results prove that SAO@H_3_PO_4_ can act as well-suited luminescent labels for plants.

**Fig. 7:**
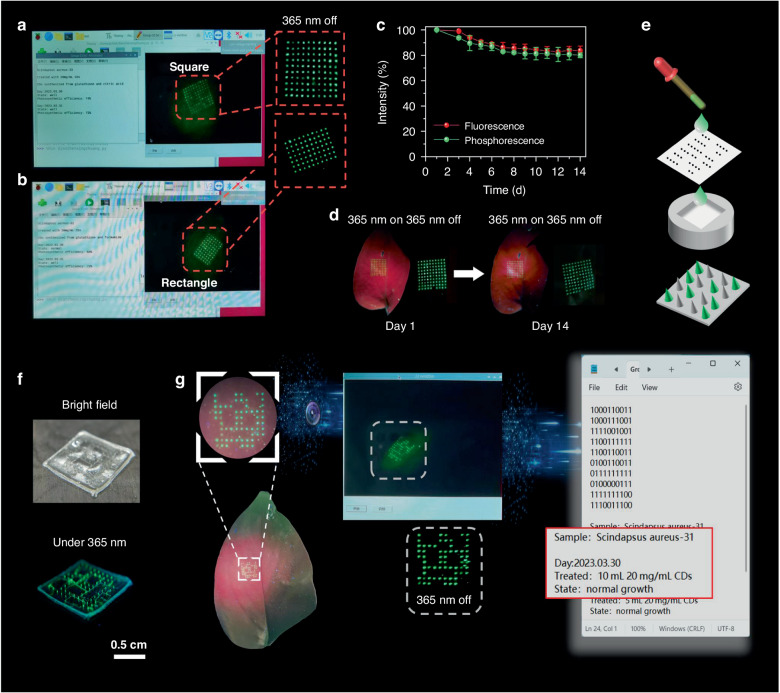
Information encoding and storage within the leaves by the plant labels. The computer interface displays square (**a**) and rectangular (**b**) plant labels collected by electronic devices and enters the corresponding information storage platform. **c** Luminescence intensities plots of the luminescent label in plants within 27 days. **d** Digital photographs of the luminescent label in the plant before and after 14 days with a 365 nm light on and off, respectively. **e** Schematic diagram of the preparation method for MNs patch containing specific arrangement combinations. **f** Under white light and 365 nm UV light, images of MNs patch which containing specific arrangement combinations. **g** Identification and information storage of plant luminescent labels, which contain specific arrangement combinations

Given that the MNs patch can efficiently deliver SAO@H_3_PO_4_ into plants with low damage, we aim to expand its functionality for creating luminescent markers within plants. To put into practice, we establish a platform for label recognition and information storage within plants. Essentially, we create different luminescent labels by employing various shapes of MNs patches, which are then used to mark plants based on their shapes. To enable label recognition and information storage, we have implemented a specially designed recognition algorithm into a high-definition camera, thereby constructing a user-friendly homemade recognition platform. The platform comprises a Raspberry Pi motherboard and a high-definition camera. The camera captures the electronic image of the label, while the Raspberry Pi executes the programmed tasks of recognizing, compiling, and presenting the information contained in the label image. With this platform, rapid and straightforward label recognition and information storage become achievable. Figure 7a, b show the square and rectangular plant labels collected by electronic devices. When exposed to a 365 nm UV lamp, the luminescent labels emit vivid green fluorescence, enabling accurate recognition of their shapes through the designed program. Even after the removal of the UV lamp, an afterglow signal persists for a period. This residual signal can be captured using cost-effective camera equipment and enhanced through signal processing in imaging. Subsequently, the designed program accurately identifies plant labels, enabling entry into the corresponding data recording platform. This process achieves low-impact implantable plant labeling and facilitates the storage of extensive information. The luminescent performance of luminescent labels embedded in plants is an important evaluation standard. Across a 27-day observation period, the luminescence intensities show a gradual decrease and maintain 78% of the original. (Fig. 7c). In Fig. 7d, luminescent images of the label retrieved from the implanted plant on the 1st and 14th days are depicted. Remarkably, even after 14 days, the label retained its original shape and distinguishable luminescence, exhibiting robust stability as a plant label.

Considering that marking plants solely by shape lacks the capacity for extensive data storage, we drew inspiration from binary information storage methods like QR codes utilized in computing.^30^ Subsequently, we developed a plant luminescent label endowed with information storage capabilities. QR codes and two-dimensional graphical labels excel in encoding and retaining information. They are composed of three positioning points, created by continuous lines, arranged in a square or circular configuration. Information storage is achieved through a configuration of black-and-white modules, providing advantages such as user-friendliness, extensive storage capacity, and error correction. As a result, QR codes have been widely adopted across diverse industries and fields. However, their application in plant injection procedures faces specific limitations. The use of continuous lines as injection points can lead to excessive plant damage, and an abundance of module points might impede natural plant growth. Therefore, this study suggests developing an array consisting of 10 × 10 fluorescent dots as plant labels. Under conditions that minimize damage, this method offers approximately 1024^10^ potential arrangement variations, ideal for extensive information storage within plants. Theoretically, by regulating the quantity of SAO@H_3_PO_4_ loaded into individual tips of the MNs patch, precise control over the entry of SAO@H_3_PO_4_ into specific needle tips becomes achievable. This allows for predetermined arrangements and combinations of luminescent and non-luminescent MNs tips within the array. Using the luminescent and non-luminescent signals of the tips as binary digits ‘1’ and ‘0’, respectively, a designated arrangement and combination of the MNs array are established. This forms the basis for conferring information storage capabilities upon the plant’s luminescent label. For example, according to the flowchart in Fig. 7e, we facilely fabricated SAO@H_3_PO_4_ loaded MNs patch with predefined arrangement and combination. Upon exposure to a 365 nm UV light (as depicted in Fig. 7f), only specific MNs tips exhibited green fluorescence. Subsequently, the loaded SAO@H_3_PO_4_ was transferred to the plant leaf surface using the aforementioned MNs patch. As illustrated in Fig. 7g, a distinct green luminescent label became visible on the plant leaf surface. The pattern and combination of its luminous sites precisely matched the arrangement and combination of the MNs tips within the MNs patch. Using the image acquisition capabilities of electronic devices alongside the label recognition functions embedded within the designed program, the luminescent and non-luminescent signals of the tips can be automatically converted into binary encoding. In Fig. 7g, after capturing the long afterglow image of the leaf, we can rapidly identify the binary encoding of the fluorescent label, this allows us to compare it with a predefined information database, enabling us to impart specific information to plants, such as the different treatments or predefined growth conditions. This strategy enables the activation of a dedicated information storage platform tailored specifically for the marked plant. The application of embedded plant luminescent labels is expected to provide new storage methods for large-scale growth-related data and further promote the development of precision agriculture.

## Discussion

In summary, we have presented a proof-of-concept for a data recording platform within plant systems. To establish this system, we used H_3_PO_4_ to encapsulate SAO and enhance its water stability while ensuring its excellent luminescent properties, laying the foundation for further in vivo applications in plants. Subsequently, customized microneedle patches were used to load and deliver the SAO@H_3_PO_4_ into plant leaves. The MNs patches made of hyaluronic acid sodium salt had sufficient hardness to easily penetrate into plant leaf tissues, and the loaded SAO@H_3_PO_4_ could stably enter the plant and maintain strong luminescent performance. Compared to normal leaves over a 14-day period, there is no significant difference in their morphological appearance, photosynthetic efficiency, and internal water transport. Using the designed plant tag recognition program and self-built in vivo plant fluorescence imaging system, the accurately identified plant luminescent tag was mapped to the corresponding data, this constructed recording platform enables information encoding and storage. Finally, this low-damage optical tag embedded in plants opens up new avenues for large-scale data storage and biosensing applications, which may promote the development of precision agriculture.

## Materials and methods

### Materials

All the chemical agents used were of analytic grade. SrCO_3_, Al(OH)_3_, Eu_2_O_3_ and Dy_2_O_3_, H_3_PO_4_ (≥85 wt% in H_2_O), and anhydrous ethanol were purchased from Shanghai Macklin Biochemical Co., Ltd. Hyaluronic acid sodium salt (mol wt 130,000–150,000) was purchased from Shanghai Picasso Technology Co., Ltd.

### Characterizations

Transmission electron microscopy (TEM) observations were performed on a Talos F200S microscope (FEI, USA). Fluorescence emission and excitation spectra and fluorescent lifetimes were recorded on a Hitachi F-7000 spectrophotometer under ambient conditions. The surface morphologies and particle sizes of the samples were analyzed by a ULTRA Plus (Zeiss, Germany) field emission scanning electron microscope (SEM). The phase of SAO was analyzed by an XD-2X/M4600 diffractometer using Cu Kα radiation (*λ* = 0.1541 nm, 40 kV, 40 mA, 10° min^−1^ from 10° to 80°). Inductively coupled plasma spectroscopy (ICP) was performed with Agilent 7700/7800 (Agilent Technologies, USA). The mechanical properties of the microneedles were tested using an INSTRON 5982 materials testing machine (INSTRON, USA). The photosynthetic activities of PS II were measured by a PAM-2500 Handy PEA chlorophyll fluorimeter (WALZ, Germany). The identification of labels utilizes a Raspberry Pi motherboard equipped with a high-definition camera.

### Synthesis and encapsulation of SAO

Based on our previously reported method, the preparation and encapsulation of SAO particles were carried out^21^. The sample was prepared by weighing out the appropriate nominal composition of Sr_0.97_Al_2_O_4_: 0.01Eu^2+^, 0.02Dy^3+^, then mixed thoroughly by ball milling for 1 h with a combination of SrCO_3_, Al(OH)_3_, Eu_2_O_3_, and Dy_2_O_3_ to ensure complete homogenization. Subsequently, the mixture was calcined at 1300 °C for 3 h under a flowing 3% H_2_–N_2_ gas-reducing atmosphere, followed by natural cooling to obtain the crude product. The calcined crude product was further ground and screened to obtain smaller particles.

Next, the obtained strontium aluminate particles were subjected to encapsulation treatment. Specifically, 2 g of strontium aluminate particles were added to 20 mL of anhydrous ethanol and stirred to form a suspension. A mixture of 10 mL anhydrous ethanol and 0.5 mL H_3_PO_4_ was prepared and thoroughly mixed. Subsequently, the solution was dropwise added into the suspension under continuous vigorous stirring, followed by aging at room temperature for 24 h. Finally, the encapsulated particles were washed three times with anhydrous ethanol and dried under vacuum to obtain the SAO@H_3_PO_4_.

### Preparation of SAO@H_3_PO_4_-loaded MNs patch

Microneedles patch was prepared using a microfabrication method^26^. In brief, SAO@H_3_PO_4_ (0.05 g) was dispersed in 1 mL of water to obtain a suspension. Hyaluronic acid sodium salt (0.25 g) was added to the suspension in three intervals with vigorous stirring using a glass rod to obtain a gel-like mixture. The mixture was then vacuum-filled into a PDMS mold under a pressure of −0.1 MPa for 10 min. After removing any bubbles on the surface, the above operation was repeated twice, and the excess mixture on the surface was removed. Next, hyaluronic acid sodium salt (0.5 g) was added to 1.5 mL of water in three portions to obtain a transparent gel-like mixture, which was evenly added into the PDMS mold to form a base. The sample was then air-dried at room temperature in a color-changing silica gel for 12 h, followed by peeling off from the mold and storing it in a dryer at room temperature.

### Leaf physiological indicators measurements

Before the measurement, the *Epipremnum aureum* was adapted in the dark overnight to balance the redox states of Photosystem II. Then, the leaves of each sample were collected to measure the chlorophyll fluorescence parameters (Table 1) by a chlorophyll fluorimeter under gradient-elevated photosynthetic active radiations (PAR) (0, 8, 36, 72, 109, 149, 203, 274, 360, 463, 597, 748, 916, 1083, 1283, 1530 and 2013 μmol m^−2^ s^−1^). More than 6 accuracy measurements were conducted on the treated *Epipremnum aureum* leaves. The *Epipremnum aureum* was not delivered with SAO@H_3_PO_4_ as a control.

**Table 1:** Definition of chlorophyll fluorescence parameters^31^

Parameters	Definition
*Y*(II)	Quantum yield of photochemical energy conversion in PS II
*Y*(NO)	Quantum yield of non-regulated non-photochemical energy loss in PS II
NPQ	Non-photochemical fluorescence quenching parameter describing regulated dissipation of excess energy, reflecting the light protection ability of plant
qP	Photochemical fluorescence quenching parameter estimating the fraction of open PS II centers based on a lake model, reflecting the photosynthetic activity
ETR	Electron transport rate in PS II

### Characterization of sap transport in plants

We chose Rhodamine B (RhB) as the coloring agent for plant sap because of its strong fluorescence emission, and the red fluorescence can be easily observed in the stem of bean sprouts. First, SAO@H_3_PO_4_ was delivered to the stem of bean sprouts using an MNs patch, followed by injection of a small amount of RhB solution at a position closer to the root using a medical needle. After incubation in deionized water for 18 h, the fluorescence signals inside the bean sprouts were recorded under 365 nm UV light. By observing the distribution of fluorescence signals, the flow of plant sap inside the plant was inferred.

### Construction of luminescent plant labels

The prepared MNs patch can be directly used for constructing plant labels, as follows: The needle tip of the MNs patch is aligned with the injection site on the plant leaf, and a certain amount of pressure is applied for 1 min to allow the needle tip to penetrate the plant leaf as much as possible and dissolve in water to release the loaded SAO@H_3_PO_4_. After injection, the MNs patch is removed, and under 365 nm excitation, the constructed luminescent plant label can be observed on the surface of the leaf.

### Construction of the luminescent plant label recognition platform

The label recognition platform consisted of a Raspberry Pi motherboard and a high-definition camera, which were used to perform functions such as image acquisition and enhancement processing, label recognition, and information storage through a designed Python program. In the label recognition platform, a 365 nm excitation light source was placed above the plants with labels to excite the strontium aluminate material in the label. A bandpass filter with a center wavelength of 520 nm was equipped in front of the camera so that only the fluorescence signal emitted by the strontium aluminate material could reach the camera for imaging.

### Fixation of the leaves for SEM imaging

Fixation of leaves was carried out by fixing samples with 2.5% glutaraldehyde and then 1% Osmium, rinsing them with phosphate buffer (pH 7.4), and dehydrating the specimen with a graded ethanol series (30%, 50%, 70%, 80%, 90%, 95%, 100%) before freeze-drying.

### MTT assay

MTT (3-(4,5-dimethylthiazol-2-yl)-2,5-diphenyltetrazolium bromide) assay was performed on HeLa cell lines. The cell viability was determined after exposure to SAO@H_3_PO_4_ at different concentrations (2, 4, 200, 1000 mg/L) for up to 48 h. After incubation, the cells were washed twice with DPBS and then incubated with MTT solution (450 μg/mL) for 4 h. After that, the culture medium was removed and then added dimethyl sulfoxide (DMSO). The mixture was then shaken for 1 min at room temperature. The optical density (OD) of the mixture was recorded at 570 nm, and the values were compared with that of control cells.

### Supplementary information

revised SI
