# Supplementary material for: Delivery of luminescent particles to plants for information encoding and storage
Source: Light Sci Appl. 2024 Aug 28;13:217. doi: 10.1038/s41377-024-01518-x (PMC11358502; doi:10.1038/s41377-024-01518-x)
Supplement: Supplementary file 1 — revised SI [file 41377_2024_1518_MOESM1_ESM.docx]

**Supplementary Information for:**

**Delivery of Luminescent Particles to Plants for Information Encoding and Storage**

Wei Li^a^, Junjie Lin^a^, Wanyi Huang^a^, Qingrou Wang^a^, Haoran Zhang^a^, Xuejie Zhang^a^, Jianle Zhuang^a^, Yingliang Liu^a^, Songnan Qu^b^, Bingfu Lei^a,c^ *

^a^ Key Laboratory for Biobased Materials and Energy of Ministry of Education, College of Materials and Energy, South China Agricultural University, Guangzhou 510642, China

^b^ Joint Key Laboratory of the Ministry of Education, Institute of Applied Physics and Materials Engineering, University of Macau, Macau 999078, China

^c^ Maoming Branch, Guangdong Laboratory for Lingnan Modern Agriculture, Guangdong Maoming 525100, China

Email: tleibf@scau.edu.cn


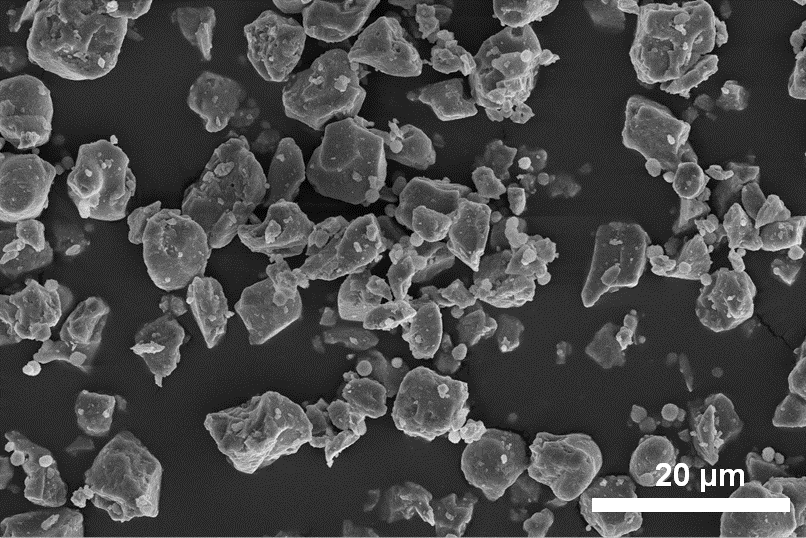


Fig. S1 SEM image of SAO@H_3_PO_4_.


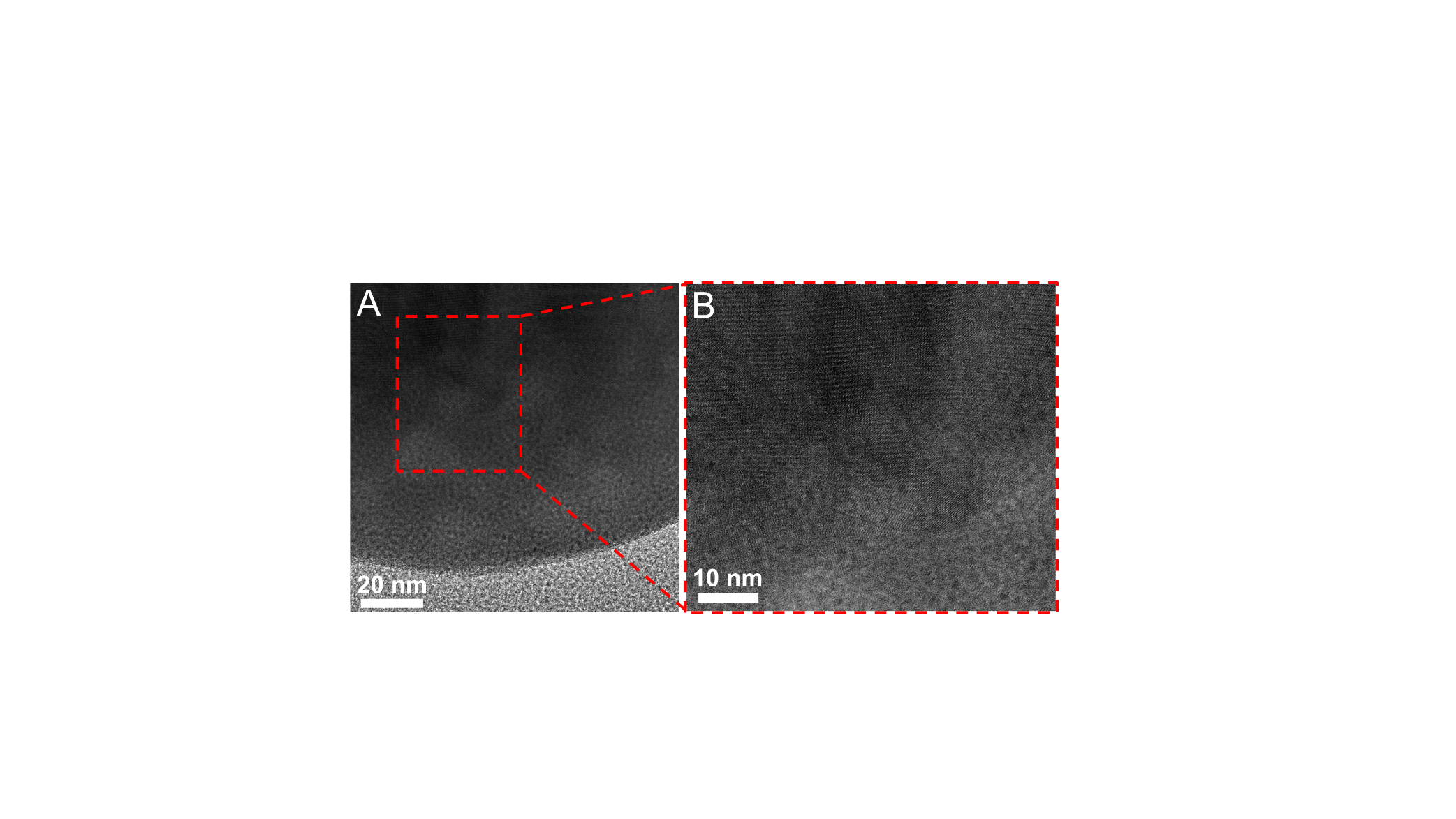


Fig. S2 (A-B) TEM images of SAO.

Fig. S3 pH values of SAO and SAO@H_3_PO_4_ suspensions in water within 7 days, respectively.


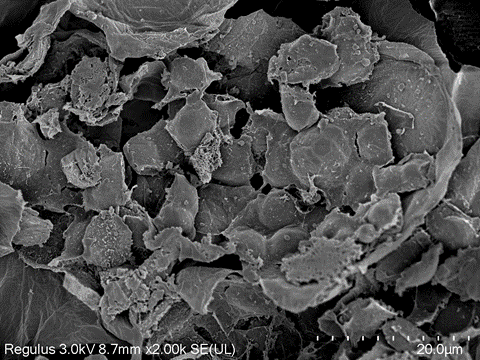


Fig. S4 SEM image of the SAO@H_3_PO_4_ injected leaf.


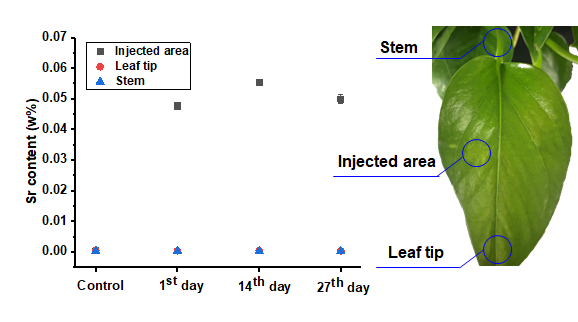


Fig. S5 Sr element content in different parts of leaves collecting from the control group, 1-day, 14-day, and 27-day luminescent labelled groups, respectively.


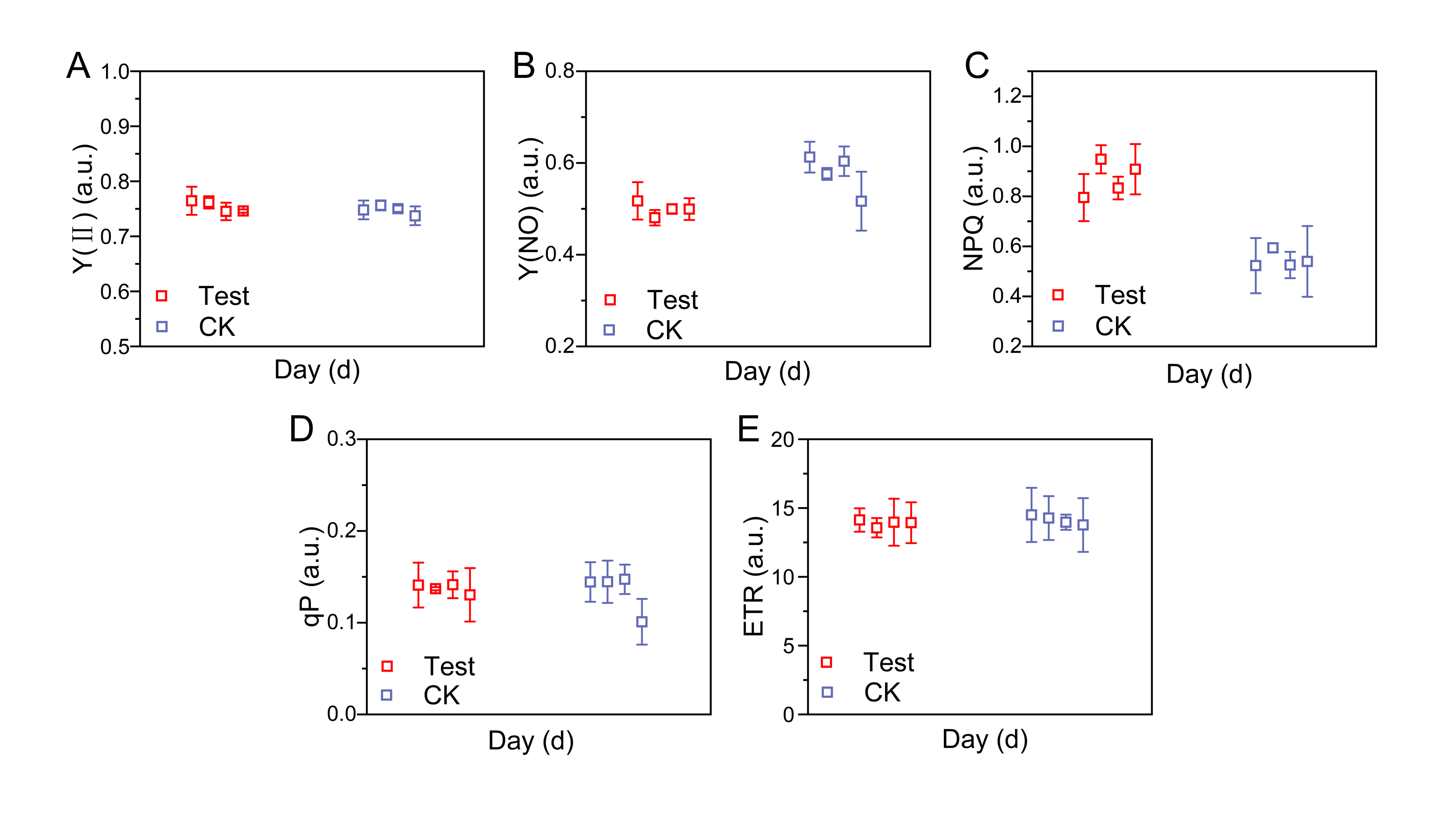


Fig. S6 Physiological indicators of leaves on days 1, 3, 5, and 7. Y(II) (A), Y(NO) (B), NPQ (C), qP (D) and ETR (E) of leaves delivered with SAP and untreated. s. Error bars correspond to standard deviation (n = 3).


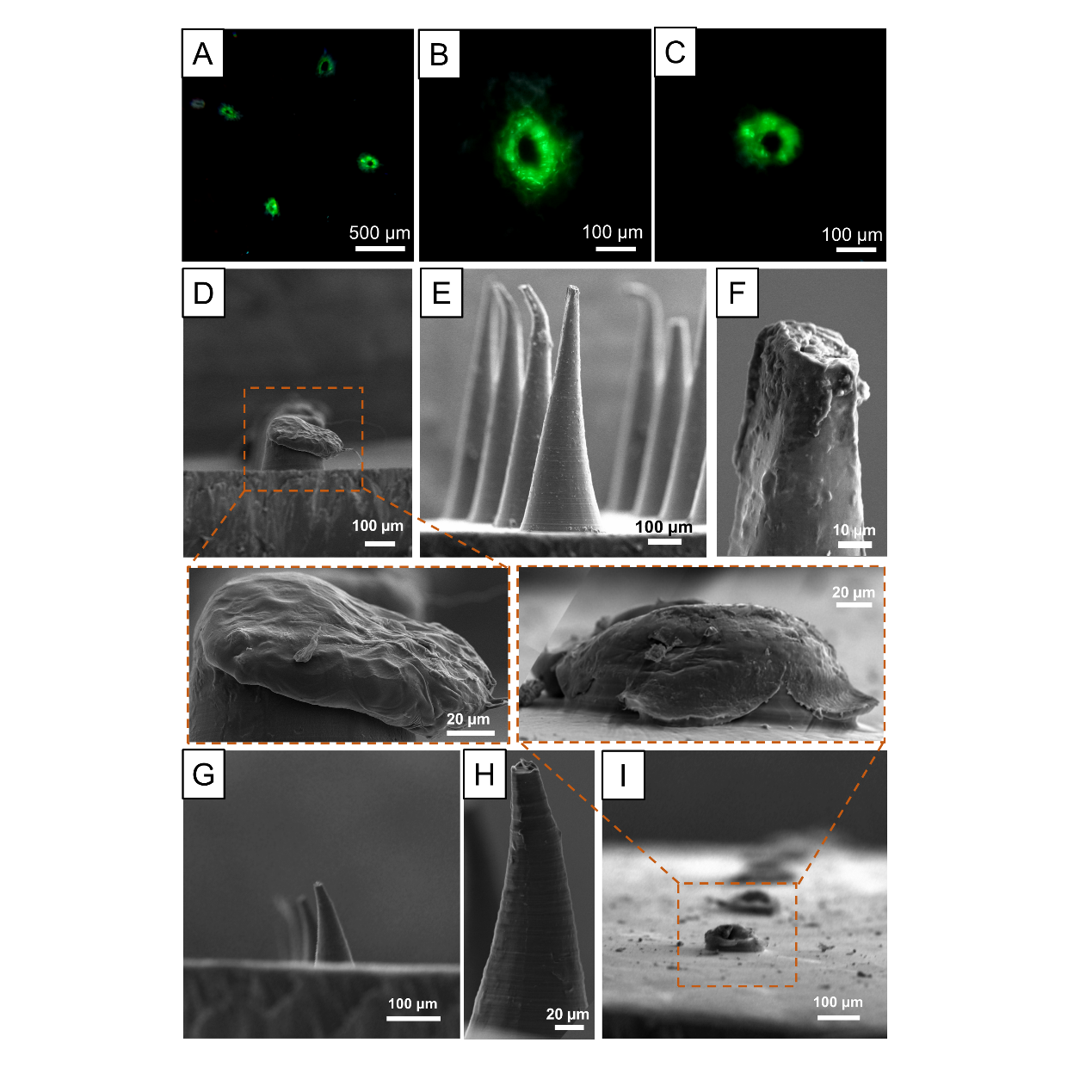


Fig. S7 Enlarged images of optical labels in plant leaves.


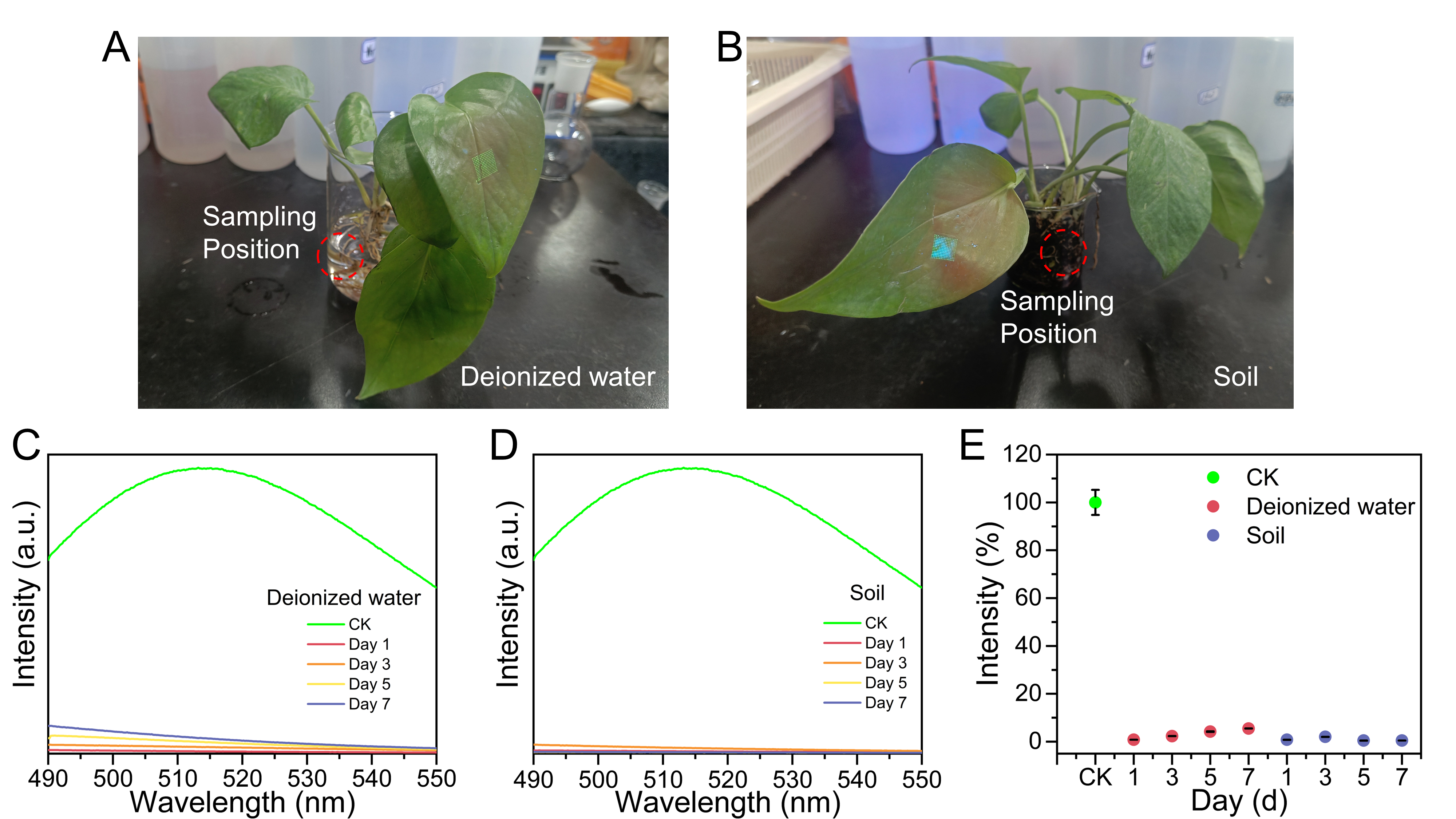


Fig. S8 Sampling location images of deionized water (A) and soil (B) environments; Fluorescence spectra of the deionized water (C) and soil (D) environments in which plants grow after injection on days 1, 3, 5, and 7; (E) Fluorescence intensity changes in the water and soil environments of plant growth after injection.


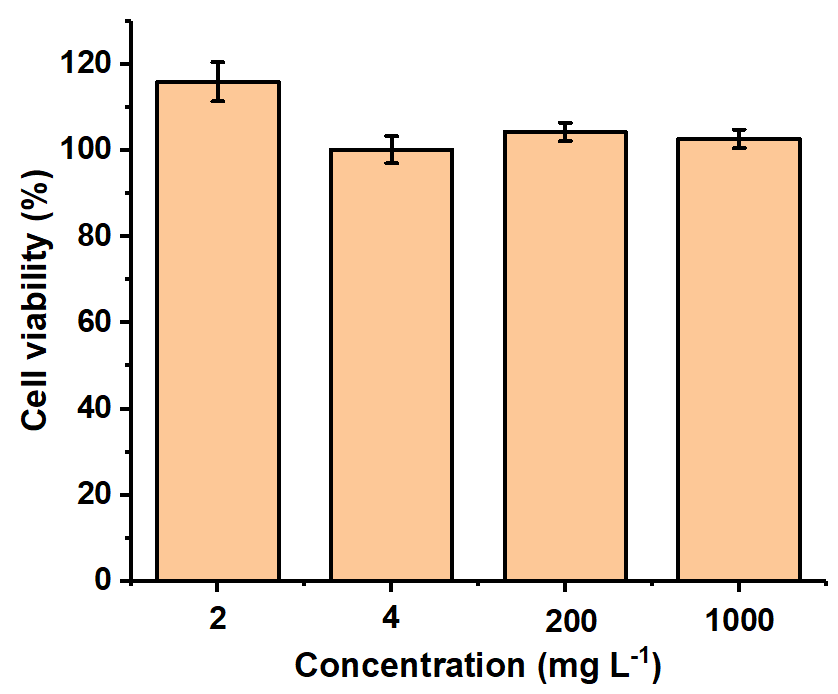


Fig. S9 MTT assay on HeLa cell line after incubating with different concentration of SAO@H_3_PO_4_.
